# Lyso-Gb3 modulates the gut microbiota and decreases butyrate production

**DOI:** 10.1038/s41598-019-48426-4

**Published:** 2019-08-19

**Authors:** John-Jairo Aguilera-Correa, Patricia Madrazo-Clemente, María del Carmen Martínez-Cuesta, Carmen Peláez, Alberto Ortiz, María Dolores Sánchez-Niño, Jaime Esteban, Teresa Requena

**Affiliations:** 1 Clinical Microbiology Department, IIS-Fundación Jiménez Díaz, UAM. Av. Reyes Católicos, 2, 28040 Madrid, Spain; 2 Department of Food Biotechnology and Microbiology, Instituto de Investigación en Ciencias de la Alimentación, CIAL (CSIC-UAM), Nicolás Cabrera, 9, 28049 Madrid, Spain; 3 Nephrology Department. IIS-Fundación Jiménez Díaz, UAM. Av. Reyes Católicos, 2, 28040 Madrid, Spain

**Keywords:** Digestive signs and symptoms, Metabolic disorders

## Abstract

Fabry disease is a rare X-linked lysosomal storage disorder resulting from deficient activity of α-galactosidase A, leading to the accumulation of glycosphingolipids such as globotriaosylsphingosine (lyso-Gb3). The gastrointestinal symptoms of this disease may be disabling, and the life expectancy of affected patients is shortened by kidney and heart disease. Our hypothesis was that lyso-Gb3 may modify the gut microbiota. The impact of a clinically relevant concentration of lyso-Gb3 on mono- or multispecies bacterial biofilms were evaluated. A complex bacterial community from the simulated transverse colon microbiota was studied using quantitative PCR to estimate different bacterial group concentrations and a HPLC was used to estimate short-chain fatty acids concentrations. We found that lyso-Gb3 increased the biofilm-forming capacity of several individual bacteria, including *Bacteroides fragilis* and significantly increased the growth of *B. fragilis* in a multispecies biofilm. Lyso-Gb3 also modified the bacterial composition of the human colon microbiota suspension, increasing bacterial counts of *B. fragilis*, among others. Finally, lyso-Gb3 modified the formation of short-chain fatty acids, leading to a striking decrease in butyrate concentration. Lyso-Gb3 modifies the biology of gut bacteria, favoring the production of biofilms and altering the composition and short-chain fatty-acid profile of the gut microbiota.

## Introduction

Fabry disease is a rare X-linked lysosomal storage disorder caused by deficient activity of α-galactosidase A. This abnormality leads to lysosomal and extralysosomal accumulation of its substrate, globotriaosylceramide (Gb3), as well as other glycosphingolipids, such as globotriaosylsphingosine (lyso-Gb3), in a variety of cell types and plasma^1,2^. Classical Fabry disease first manifests in childhood, but more limited symptoms are observed in late-onset Fabry disease^3,4^. The symptoms are more severe in males but are also present in females^5^. Childhood disease is characterized by neuropathic pain, gastrointestinal symptoms, angiokeratoma, and hypohidrosis followed by development of proteinuric nephropathy, leading to end-stage renal disease requiring dialysis at a mean age of 40 years, left ventricular hypertrophy, arrhythmia, and stroke^1,6^. Current specific therapy includes replacement of the missing enzyme through biweekly parenteral administration of agalsidase and oral therapy with the chaperone migalastat^4,7^.

Most patients with Fabry disease report gastrointestinal symptoms such as abdominal pain, diarrhea, constipation, nausea, vomiting, and early satiety. These symptoms may be severe and negatively impact quality of life and body weight, potentially leading patients to undergo unnecessary surgical interventions^8–11^. While agalsidase therapy may improve gastrointestinal symptoms, this not always is the case, and there is a pressing need to better understand the pathogenic mechanisms of these symptoms^4,5,7,12^. Currently, there are two dominant hypotheses as to the mechanisms of the gastrointestinal symptoms reported in Fabry disease: dysfunction of autonomic neurons controlling gut motility^13^ on the one hand and vascular dysfunction and/or ischemia due to intestinal smooth-muscle or endothelial cell injury^14^ on the other. These mechanisms lead to a rapid gut transit time, impaired peristalsis, gastroparesis and intestinal stasis, bacterial overgrowth, and nutrient malabsorption^15^. Thus, gut-bacterial dysbiosis is thought to contribute to the gastrointestinal symptoms associated with Fabry disease, though until now it was thought to be secondary to stasis and dysmotility^15^. An altered gut microbiota may contribute to the pathogenesis and symptoms of both gastrointestinal and systemic diseases^16–18^. Indeed, gut biofilm–forming bacteria have been implicated in gastrointestinal disease^19^. Additionally, an altered microbiota may release uremic toxins or their precursors, which accelerate the progression of chronic kidney disease and cardiovascular disease, both key consequences of Fabry disease^20–22^, or may impair the release of protective molecules that modulate the inflammatory and immune responses, among others^23^.

We hypothesized that the metabolic derangement that takes place in Fabry disease may directly modify the biology of gut bacteria. Globotriaosylsphingosine (lyso-Gb3) is a deacylated form of Gb3 considered to be a diagnostic marker for Fabry disease^24–26^. Plasma lyso-Gb3 levels may increase up to several hundred-fold over normal control values, as compared to the 2-fold increase seen in serum Gb3. Lyso-Gb3 is more hydrosoluble than Gb3, is not trapped inside lipoproteins, and has been reported to contribute to the pathogenesis of kidney, vascular, and neuronal injury^24,27–30^. A dramatic increase in lyso-Gb3 concentrations was noted at tissue level in the liver and intestine of Fabry mice, clearly exceeding plasma levels^24^. These findings may suggest the existence of a “secret road”^31^: where lyso-Gb3 is secreted from the body via bile. Consequently, lyso-Gb3 may influence the gut microbiota and contribute to gastrointestinal symptoms or other manifestations of Fabry disease. Therefore, we aimed to evaluate the impact of lyso-Gb3 on intestinal bacteria in increasingly complex *in vitro* models. These included biofilm development in individual bacterial species; four-species biofilm, and a complex transverse colon microbiota pool sample from a dynamic human gut simulator. The results suggest that lysoGb3 may directly modify the microbiota composition as well as its secreted metabolites, potentially leading to systemic effects.

## Methods

### Bacteria

Five collection and 10 clinical strains were used. The collection strains supplied by American Type Culture Collection (ATCC) (Manassas, Virginia, USA) were *Bacteroides fragilis* ATCC 25285, *Clostridium perfringens* ATCC 13124, *Enterococcus faecalis* ATCC 29212, *Escherichia coli* ATCC 25922, and *Klebsiella pneumoniae* ATCC 23357. Furthermore, two strains of each species isolated from patient samples were used (Table 1). All patient strains were isolated and identified in the clinical microbiology department of the University Hospital Fundación Jiménez Díaz of Madrid (Spain). All strains were stocked frozen at −80 °C until the experiments were performed.

**Table 1:** Origin of clinical bacterial strains used in this study.

Species	Name	Sample	Gender	Age (years)
*E. faecalis*	Ef1	Wound exudate	Female	32
	Ef2	Double-J catheter	Female	85
*E. coli*	Ec1	Ulcer	Female	82
	Ec2	Urine	Female	8
*K. pneumoniae*	Kp1	Urine	Female	60
	Kp2	Urine	Female	59
*B. fragilis*	Bf1	Drainage liquid	Female	50
	Bf2	Wound exudate	Male	55
*C. perfringens*	Cp1	Skin exudate	Female	54
	Cp2	Bile	Male	68

### Monospecies biofilm formation

Biofilm studies were performed in 96-well plates (Thermo Fisher Scientific, Waltham, Massachusetts, USA). To do this, a final concentration of 500 nM of lyso-Gb3 (Sigma-Aldrich, St. Louis Missouri, USA) was added to 10^6^ colony-forming units (CFU)/mL of each strain inoculated in 200 µL tryptic soy broth (TSB; BD, Franklin Lakes, New Jersey, USA) supplemented with 1% glucose (Sigma-Aldrich), and the bacteria were incubated at 37 °C in 5% CO_2_ atmosphere for 24 h^32^. This lyso-Gb3 concentration was chosen since it is clinically relevant: in human and murine Fabry disease, plasma lyso-Gb3 may reach values of 400–600 nM, and in mice, these values were shown to be even higher in the liver and duodenum (10,900 and 4,100 nmol/g, respectively)^24^. After incubation and medium removal, samples were washed three times with 200 µL sterile 0.9% NaCl (B. Braun, Melsungen, Germany). Then, 190 µL of TSB+ 1% glucose plus 10 µL of alamarBlue® (BIORAD, California, USA) were added and incubated at 37 °C for 60 min^33^. After incubation, fluorescence was measured at 560 nm excitation wavelength and 590 nm emission wavelength to estimate the bacterial concentration in the biofilm^33^. All experiments were performed in triplicate (n = 24 per strain, 8 wells per replica).

### Multispecies biofilm formation

Four representative strains of the previously assayed bacterial species were chosen and mixed (10^6^ CFU/mL of *E. coli* ATCC 25922 and *K. pneumoniae* ATCC 23357, and 10^8^ CFU/mL of *B. fragilis* ATCC 25285 and *C. perfringens* ATCC 13124) and incubated for 24 h in TSB+ 1% glucose with or without 500 nM lyso-Gb3 in anaerobic conditions. After incubation, each well was washed twice with 0.9% NaCl and sonicated for 5 min using an Ultrasons-H 3000840 low-power bath sonicator (J. P. Selecta, Barcelona, Spain) at 22 °C^34^. The concentration of bacteria in the biofilm was then estimated by applying the drop plate method^35^. *E. coli* ATCC 25922 and *K. pneumoniae* ATCC 23357 were quantified on chromID® CPS® Elite agar (Biomeriéux, Marcy-l'Étoile, France) in aerobic conditions, *B. fragilis* was quantified on Schaedler agar supplemented with neomycin and vancomycin (Biomeriéux), and counts of *C. perfringens* were conducted in 5% lamb’s blood agar supplemented with colistin and nalidixic acid (Biomeriéux) in anaerobic conditions. The experiment was carried out in 5 wells of 96-well plates in a volume of 200 μL/well and was repeated five times (n = 25 per species).

Multispecies biofilms were analyzed using a Leica DM IRB confocal laser-scanning microscope (Wetzlar, Lahn-Dil, Germany)^36^ in hydrophobic uncoated sterile 2-by-4–wells plates (ibidi GmbH, Munich, Bavaria, Germany) after staining with Live/Dead BactLight© stain (Thermo Fisher) according to manufacturer instructions.

### Human gut microbiota

Dynamic multistage gut simulators are relevant for *in vitro* microbial ecological studies since they allow differentiation of colon region-specific populations originated from human stool samples^37^. We investigated the impact of lyso-Gb3 on the gut microbiota obtained from the simulated transverse colon suspension. A 50-mL colon-microbiota sample was centrifuged (10,000 × *g* for 10 min) and the pellet was covered with glycerol, snap-frozen in liquid nitrogen and stored at −80 °C until the experiment was performed. For experiments, the microbiota sample was suspended in 50 mL of a previously described growth medium^37^ supplemented with 2 g/L dehydrated purified ox-bile (Sigma-Aldrich) and buffered to pH 6.5–7.0 using a carbonate-phosphate buffer (9.240 g/L NaHCO_3_, 7.125 g/L Na_2_HPO_4_•12H_2_O, 0.470 g/L NaCl, 0.450 g/L KCl, 0.070 g/L CaCl_2_•12H_2_O, and 0.1 g/L MgCl_2_•6H_2_O) following the procedures of Durand *et al*.^38^. The suspension obtained was used to inoculate (1%) fresh buffered growth medium and incubated at 37 °C in the presence or absence of 500 nM lyso-Gb3 in an anaerobic chamber with 90% N_2_, 5% CO_2_, and 5% H_2_ atmosphere (Bactron II, Sheldon Manufacturing, Sunnyvale, California, USA). After incubation for 24 h, samples were centrifuged at 13,000 × g for 5 min, and the supernatant and pellet were stored at −20 °C for further analyses. All experiments were performed in triplicate (n = 3).

### DNA extraction and purification

Microbial DNA was extracted as described by Moles *et al*.^39^. Briefly, the pellet from the colon microbiota culture was resuspended in 500 μL of 200 mM Tris–HCl pH 7.5, 0.5% SDS, 25 mM EDTA, 250 mM NaCl, and 3 M sodium acetate, and then incubated with 20 mg/mL lysozyme and 10 mg/mL RNAase (Sigma-Aldrich). Bacterial lysis was completed by mixing with glass beads. DNA was extracted with phenol/chloroform/isoamyl-alcohol, precipitated by adding 0.6 volumes of isopropanol and then resuspended in DNase, RNase free water (Sigma-Aldrich). The DNA yield was measured using a NanoDropH ND-1000 UV spectrophotometer (Thermo Fisher).

### Quantitative PCR (qPCR)

Quantitative microbiological analysis of samples was carried out in qPCR experiments analyzed using SYBR® green methodology in a ViiA7 Real-Time PCR System (Life Technologies, Carlsbad, CA, USA). Primers, amplicon size, and annealing temperature for *Akkermansia*, *Bacteroides*, *Bifidobacterium*, *Enterobacteriaceae*, *Faecalibacterium*, *Lactobacillus*, *Enterococcus*, *Prevotella*, *Roseburia, Blautia coccoides-Eubacterium rectale* Cluster XIVa, *Ruminococcus* Cluster IV, and *Clostridium leptum* subgroup specific cluster IV have been described previously^40^. For the analysis of *B. fragilis* and *Bilophila* we used the primers and PCR conditions described by Sjögren *et al*.^41^ and Baldwin *et al*.^42^, respectively. DNA from *E. coli* DH5α, *L. plantarum* IFPL935, *Enterococcus faecalis* IFPL 382, *Bifidobacterium breve* 29M2, and *B. fragilis* DSM2151 were used to quantify total bacteria and *Enterobacteriaceae*, *Lactobacillus*, *Enterococcus*, *Bifidobacterium*, and *Bacteroides* and *B. fragilis*, respectively. For all other groups analyzed, samples were quantified using standards derived from targeted cloned genes using the pGEM-T cloning vector system kit (Promega, Madison, Wisconsin, USA) as described previously^37^.

### Short-chain fatty-acid (SCFA) determination

The supernatant from the colon microbiota culture was filtered and 0.2 μL were injected into an HPLC system (Jasco, Tokyo, Japan) equipped with a UV975 detector and automatic injector^37^. SCFA were separated using a Rezex ROA Organic Acids column (300 × 7.8 mm) (Phenomenex, Macclesfield, UK) thermostated at 50 °C following the method described by Sanz *et al*.^43^. The mobile phase had a linear gradient of 0.005 M sulphuric acid in HPLC grade water, and the flow rate was 0.6 mL/min. The elution profile was monitored at 210 nm and peak identification was carried out by comparison between retention times and standards. ChromNAV data system software (Jasco) was used for data acquisition and processing. Calibration curves of acetic, butyric, formic, lactic and succinic acid were built up in the range concentration of 1 to 100 mM.

### Statistical analysis

Statistical analyses were performed using Stata Statistical Software, Release 11 (StataCorp, College Station, Texas, USA). Prior to performing statistical calculations, the normality of each series of data was checked with the Shapiro-Wilk test. Normally distributed data were presented as mean and standard deviation and compared using the unilateral Student’s *t*-test. When the distribution was not normal, data were represented as median and interquartile range, and the one-sided Wilcoxon signed-rank test was applied. A level of statistical significance of *P* < 0.05 was considered significant.

## Results

### Impact of lyso-Gb3 on monospecies biofilm formation

**Figure 1:**
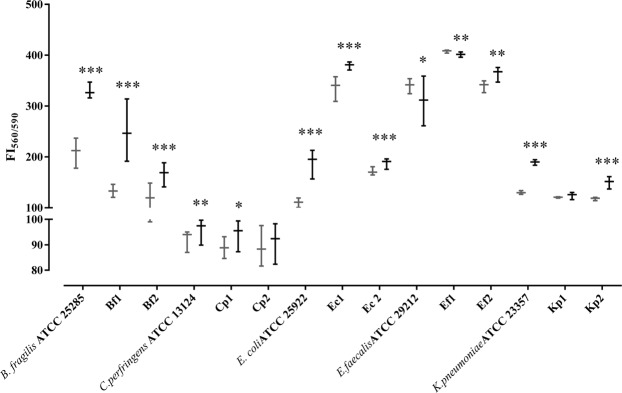
Impact of lyso-Gb3 on monospecies biofilm formation. Fluorescence intensity (FI) (x1000) of each bacterial-strain biofilm in presence (black) or absence (gray) of lyso-Gb3. The following represent strains derived from clinical isolates (Table 1): *E. faecalis* (Ef1, Ef2), *E. coli* (Ec1, Ec2), *K. pneumoniae* (Kp1, Kp2), *B. fragilis* (Bf1, Bf2), and *C. perfringens* (Cp1, Cp2). The whiskers represent the interquartile range. *p-value < 0.05,  **p-value < 0.01, and ***p-value < 0.001 for Wilcoxon signed-rank test.

Microbial biofilms containing potential pathogens are regarded as a tipping point between healthy and diseased states in the gut mucosa^44^. Thus, we first explored the impact of lyso-Gb3 on biofilm formation by different individual strains, and these results are represented in Fig. 1. All strains except two (clinical strains *K. pneumoniae* Kp1 and *C. perfringens* Cp2) significantly modified their biofilm formation in the presence of lyso-Gb3. Most strains significantly increased biofilm formation in the presence of lyso-Gb3, and only two (*E. faecalis* ATCC 29212 and Ef1) significantly but marginally decreased their biofilm formation. Thus, the most consistent results were obtained for *E. coli* and *B. fragilis*, in which all three strains tested significantly increased the biofilm formation for each species.

### Impact of lyso-Gb3 on multispecies biofilm formation

**Figure 2:**
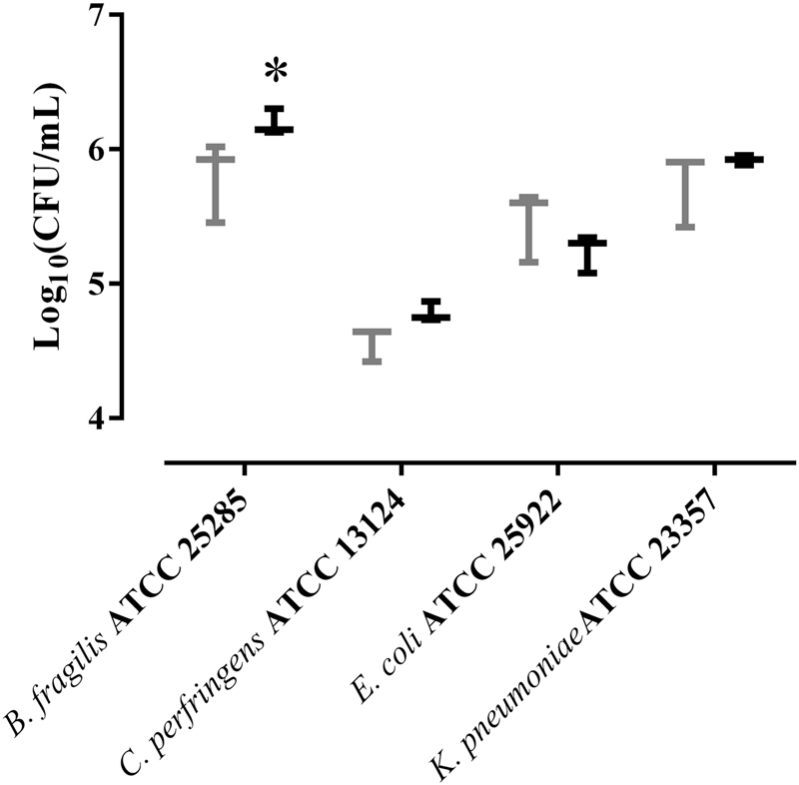
Impact of lyso-Gb3 on multispecies biofilm formation. Bacterial concentration (log CFU/mL) of each bacterial strain in a multispecies biofilm in the presence (black) or absence (gray) of lyso-Gb3. The whiskers represent the interquartile range. *p-value < 0.05 for Wilcoxon signed-rank test.

**Figure 3:**
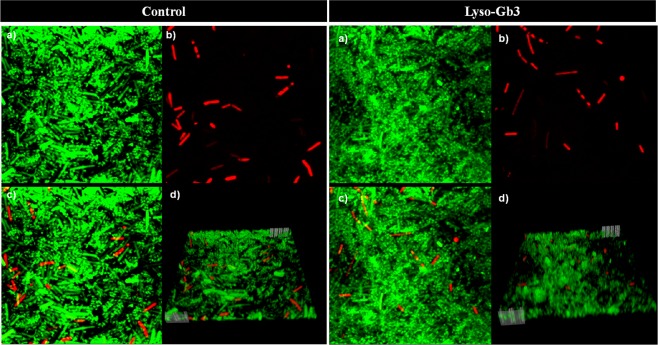
Representative confocal photograph of a multispecies biofilm in the presence or absence of lyso-Gb3 using Live/Dead BactLight^TM^. (**a**) Live bacteria in green. (**b**) Dead bacteria in red. (**c**) Superposition of live and dead bacteria. (**d**) Three-dimensional representation of each biofilm.

We then studied the impact of lyso-Gb3 on the formation of polymicrobial biofilms containing potential pathogens. The results of multispecies (*E. coli* ATCC 25922, *K. pneumoniae* ATCC 23357, *B. fragilis* ATCC 25285, *C. perfringens* ATCC 13124) biofilm formation are shown in Fig. 2, and a representative three-dimensional representation is shown in Fig. 3. Only *B. fragilis* ATCC 25285 numbers significantly increased in presence of lyso-Gb3 in a multispecies biofilm model (p = 0.0236). This result is consistent with the monospecies biofilm studies.

### Impact of lyso-Gb3 on complex bacterial communities

Since the bacterial populations of the gut microbiota are even more complex, we explored the impact of lyso-Gb3 on the composition and metabolite output of the simulated human transverse colon microbiota. Samples were analyzed for 16S rRNA qPCR quantification of bacteria (pellet) and for SCFA formation (supernatant). The targeted bacterial groups represent the predominant Gram-positive bacteria belonging to clostridial clusters XIVa and IV (Firmicutes) and Gram-negative bacteria related to Bacteroidetes. Other genera such as *Lactobacillus*, *Bifidobacterium* and *Akkermansia* are commonly health-related bacteria. Specific quantification of *Bilophila* was targeted for its correlation with intestinal discomfort^45,46^. The qPCR assay showed that lyso-Gb3 modified several groups of bacteria, as shown in Table 2. Most bacterial groups evaluated significantly modified their concentration in presence of 500 nM lyso-Gb3 with respect to controls, the exceptions being *Bilophila*, *Faecalibacterium*, and *Ruminococcus*. Those bacterial groups with significantly higher concentrations under lyso-Gb3 were Enterobacteriaceae, *Enterococcus*, and *Prevotella*. The largest effect of lyso-Gb3 to increase bacterial counts was observed for *B. fragilis*, as with single bacterial species and multispecies biofilm experiments. By contrast, the concentration of *Akkermansia*, *Bacteroides*, *Bifidobacterium*, *C. leptum*, *B. coccoides*-*E. rectale*, and *Lactobacillus* was significantly lower under lyso-Gb3 than in controls.

**Table 2:** Impact of lyso-Gb3 on bacterial counts in a transverse colon microbiota sample.

Bacterial group	Baseline	Control 24 h	500 nM lyso-Gb3 24 h	p-value*
*Akkermansia*	6.74 ± 0.06	6.22 ± 0.10	5.91 ± 0.10	<0.001
*Bacteroides*	6.50 ± 0.22	7.62 ± 0.13	6.73 ± 0.10	<0.001
*B. fragilis*	3.78 ± 0.02	1.55 ± 0.35	5.09 ± 0.18	<0.001
*Bifidobacterium*	4.40 ± 0.06	4.09 ± 0.05	3.92 ± 0.10	0.003
*Bilophila*	6.75 ± 0.04	7.94 ± 0.09	7.96 ± 0.07	0.328
*Clostridium leptum*	5.26 ± 0.05	5.20 ± 0.16	4.85 ± 0.05	0.001
*Blautia coccoides-Eubacterium rectale*	6.80 ± 0.06	7.07 ± 0.02	6.81 ± 0.12	0.002
*Enterobacteriaceae*	6.76 ± 0.07	8.70 ± 0.08	8.95 ± 0.14	0.002
*Enterococcus*	6.19 ± 0.05	7.74 ± 0.17	8.06 ± 0.07	0.002
*Faecalibacterium*	7.88 ± 0.10	7.48 ± 0.06	7.53 ± 0.16	0.276
*Lactobacillus*	5.42 ± 0.10	4.18 ± 0.05	3.91 ± 0.06	<0.001
*Prevotella*	2.79 ± 0.28	3.70 ± 0.09	4.34 ± 0.32	0.002
*Roseburia*	3.72 ± 0.15	3.11 ± 0.08	3.33 ± 0.16	0.006
*Ruminococcus*	3.98 ± 0.07	3.09 ± 0.14	2.94 ± 0.18	0.074

Mean ± SD of quantitative PCR counts (log copy number/mL) for the different microbial groups analyzed.

*p-values for Student’s t-test between control 24 h and 500 nM lyso-Gb3 24 h.

SCFA determination showed that lyso-Gb3 could also modify SCFA production by the gut microbiota, as shown in Table 3. The most remarkable effect observed was the significantly lower formation of butyric acid in lyso-Gb3-exposed samples than in controls. In addition, an unknown acidic compound (peak retention time 33.4) was only observed in the samples incubated with lyso-Gb3 (results not shown).

**Table 3:** Impact of lyso-Gb3 on short chain fatty acid (SCFA) concentration in a transverse colon microbiota sample.

SCFA	Baseline	Control 24 h	500 nM lyso-Gb3 24 h	p-value*
Acetic acid	1.21 ± 0.08	29.55 ± 2.11	31.28 ± 2.03	0.101
Butyric acid	ND	1.48 ± 0.56	0.68 ± 0.20	0.008
Formic acid	9.01 ± 0.24	18.46 ± 1.59	15.96 ± 0.11	0.006
Lactic acid	ND	7.21 ± 0.86	7.29 ± 0.42	0.428
Succinic acid	12.25 ± 0.7	14.1 ± 0.80	15.36 ± 0.20	0.006

SCFA concentrations in mM; expressed as mean ± SD.

*p-values for Student’s t-test between control 24 h and 500 nM lyso-Gb3 24 h. ND: not detected.

## Discussion

The pathophysiology of gastrointestinal symptoms in Fabry disease is complex and multifactorial, though the fact that these symptoms stem from Gb3 and lyso-Gb3 accumulation within intestinal tissues is now widely accepted^15^. Here, we have shown that lyso-Gb3 may directly modify microbiota composition, potentially contributing to the gastrointestinal and systemic symptoms of Fabry disease.

The gut microbiota is a collection of archaea, bacteria, and eukarya that have evolved over thousands of years to form a symbiotic relationship with human hosts^47^. These microbial populations influence metabolic, immune and defense systems in the intestine and consequently, human health^17,48–50^. The bacterial phyla representatives of the human gut microbiota are Bacteroidetes, Firmicutes, Actinobacteria, Proteobacteria and Verrucomicrobia; additionally, the genera *Faecalibacterium*, *Bifidobacterium*, *Roseburia*, *Ruminococcus*, *Bacteroides*, *Prevotella*, *Akkermansia* and *Oscillospira* represent common core bacteria in the Western adult population^51^. These gut bacteria are present in both planktonic and biofilms states which may be associated with luminal material or mucosal surfaces^44,52–56^, such as gut biofilms commonly associated with disease^44,57^. According to our results, the presence of lyso-Gb3 at clinically relevant concentrations significantly favored biofilm development by *B. fragilis* and *E. coli* and by some strains of *C. perfringens*, *E. faecalis*, and *K. pneumoniae*. Since gut biofilms are composed of multiple microbial species^17,18,53,58,59^, the most realistic *in vitro* approach would be to simultaneously develop a multispecies biofilm using several intestinal bacterial species. Indeed, the only species significantly favored by lyso-Gb3 when this approach was taken was *B. fragilis*.

The dynamic and complex interactions of colonic bacteria have been nearly reproduced *in vitro* by using laboratory simulators of the human gut microbiome^37,60,61^. The transverse-colonic compartment is considered to hold most of the representative colonic bacterial groups^37,61^, so we adopted this approach to increase the complexity of our studies. In view of our results, all evaluated groups of bacterial underwent modified growth to a greater or lesser degree in the presence of lyso-Gb3. The most striking modification was observed in *B. fragilis*, as the concentration of this bacteria strain increased almost 1.5 log-fold during 24 h incubation in the presence of lyso-Gb3, whereas no growth was observed in the control. This positive impact reflected the biofilm study results. *B. fragilis* is a two-faced gut symbiotic bacteria^62^. On the one hand, lipopolysaccharide A and other polysaccharides from *B. fragilis* stimulate the development of regulatory T cells which, in turn, switch off inflammatory T cells, thus offering protection from local or systemic inflammatory processes^59,63^. On the other hand, *B. fragilis* strains may release an enterotoxin or *B. fragilis* toxin (BFT), which is associated with diarrhea in young animals^64^, children^64,65^, and adults^66^. BFT has been linked to colorectal adenoma, polyps, and cancer in experimental animals and humans^62,67–71^. Additionally, *B. fragilis* biofilm development has been associated with inflammatory bowel diseases^52,67^. Thus, changes in *B. fragilis* biology induced by lyso-Gb3 could potentially be linked to the gut inflammation and colonic polyps described in Fabry disease^72,73^.

SCFA are fermentation products of bacterial microbiota^58^. Acetic, propionic and butyric acids can serve as an energy source to human intestine epithelium^17^. The most striking impact of lyso-Gb3 observed in this study was to decrease the butyrate formation by almost 50%. This may be caused by the decrease in Firmicutes (including *C. leptum* and *B. coccoides*-*E. rectale* groups) counts, as Firmicutes are the main producers of butyrate^17,58,74^, via cross-feeding of acetate and lactate^74^. However, we cannot exclude a more direct impact of lyso-Gb3 on butyrate metabolism. Butyrate has several beneficial effects, including protection against colorectal cancer, chronic kidney disease, and left ventricular hypertrophy, the latter two being hallmarks of Fabry disease^18,20,75^. Butyrate also inhibits histone deacetylases (HDACs) and has anti-inflammatory properties^23,76^. Chronic low-level inflammation is thought to contribute to Fabry-disease severity by enhancing the activity of upstream enzymes, such as Gb3 synthase, which increases the availability of accumulated metabolites such as Gb3^77^. HDAC inhibitors modify the epigenetic regulation of gene transcription and, as butyrate itself, have been beneficial in kidney disease^20,78–81^.

Gastrointestinal symptoms may severely compromise the quality of life of Fabry patients and lead them to undergo unnecessary surgical interventions^15^. While bacterial overgrowth in Fabry disease was observed in one 40-year-old patient and has been cited as a potential contributor to gastrointestinal symptoms ever since, we found no reports in which gut microbiota composition was assessed using modern techniques, and available data rely on non-specific breath tests^14,82^. Thus, one limitation of the present study is the lack of comparative clinical information on gut microbiota between Fabry patients and healthy controls. Given that Fabry is a rare disease, a multicenter collaborative study would be needed to address this. One of the strengths of this research is the fact that we studied a clinically relevant lyso-Gb3 concentration and observed consistent results across three different independent experimental settings for *B. fragilis*. The negative impact of lyso-Gb3 observed on butyrate, a SCFA with beneficial properties for cardiac hypertrophy and chronic kidney disease, lends further potential clinical relevance to our findings. In this regard, the hypothesis raised in the present study regarding a microbiota connection between glycolipid accumulation and potential local or systemic consequences are very much in line with recent developments in the interaction between gut microbiota and disease^48,76,83–85^. This experimental work has several limitations. First, only one clinically relevant lyso-Gb3 concentration was tested in several experimental conditions. Second, we cannot exclude that other glycosphingolipids associated to this Fabry disease such as Gb3 or lactosylceramide^24^, which could theoretically be present in bile^31^ or present within sloughing enterocytes^24,86^, also modulate the microbiota. However, Gb3 deposits have not been observed in enterocytes, likely due to the short half-life of these cells, since longer lived cells (neurons, podocytes, cardiomyocytes) are the ones with the largest burden of deposits^87^.

**Figure 4:**
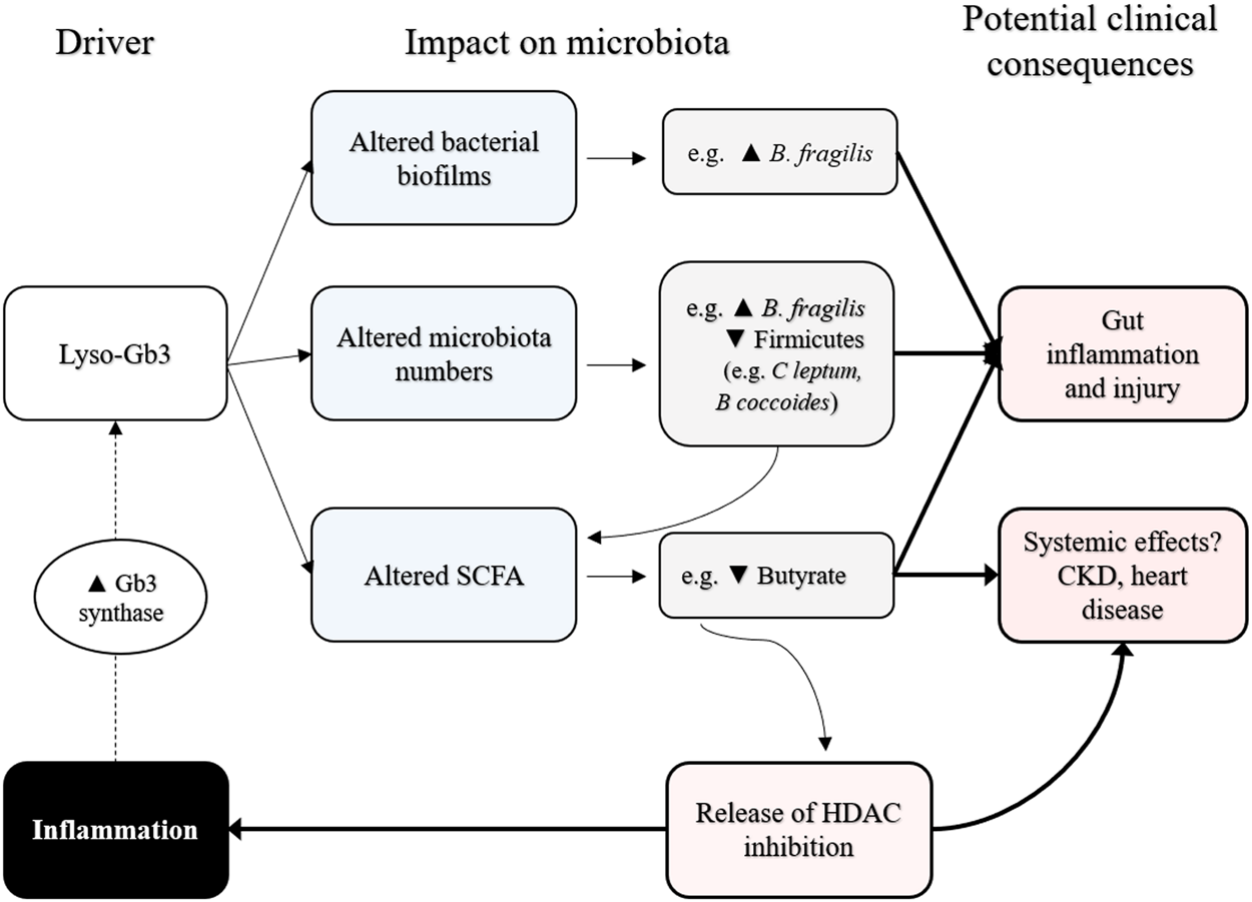
Summary of research findings and working hypothesis for possible clinical implications. At clinically relevant concentrations, lyso-Gb3 modified the biofilm-forming properties and counts of diverse bacteria. Most notably, lyso-Gb3 consistently increased *B. fragilis* biofilm formation or counts in three different, independent experimental settings. Additionally, it led to decreased production of butyrate and an altered pattern of other short-chain fatty acids (SCFA). Experimental data from the literature have linked low butyrate levels to gastrointestinal manifestations and kidney and heart disease, which are also found in Fabry disease. Thus, butyrate has been reported to have anti-inflammatory properties attributable to its ability to inhibit histone deacetylases (HDACs)^23,79–81^. In this regard, we hypothesize that lyso-Gb3-induced lowering of butyrate levels may release the inhibition of butyrate on HDACs and favor systemic inflammation. Inflammation, in turn, is known to increase the activity of Gb3 synthase^77^, thus increasing the synthesis of metabolites such as Gb3 that accumulate in Fabry disease and potentially increasing the severity of kidney and heart disease.

In conclusion, globotriaosylsphingosine (lyso-Gb3) may modify the growth and biofilm-forming capacity of intestinal bacteria as well as the SCFA formation pattern of healthy gut microbiota. To our knowledge, this is the first study to reveal a possible direct relationship between metabolites accumulated in Fabry disease, such as lyso-Gb3, and the gut microbiota, potentially causing an impact on the gastrointestinal and even systemic symptoms of Fabry disease, opening a whole new field of Fabry research (Fig. 4). However, more in-depth microbiologic studies are necessary to understand the molecular mechanisms linking lyso-Gb3 to bacterial metabolism, and further work exploring the *in vivo* clinical and therapeutic consequences of this observation would be required.
