# Publisher Correction: Lyso-Gb3 modulates the gut microbiota and decreases butyrate production

**DOI:** 10.1038/s41598-020-62302-6

**Published:** 2020-03-20

**Authors:** John-Jairo Aguilera-Correa, Patricia Madrazo-Clemente, María del Carmen Martínez-Cuesta, Carmen Peláez, Alberto Ortiz, María Dolores Sánchez-Niño, Jaime Esteban, Teresa Requena

**Affiliations:** 1 Clinical Microbiology Department, IIS-Fundación Jiménez Díaz, UAM. Av. Reyes Católicos, 2, 28040 Madrid, Spain; 2 Department of Food Biotechnology and Microbiology, Instituto de Investigación en Ciencias de la Alimentación, CIAL (CSIC-UAM), Nicolás Cabrera, 9, 28049 Madrid, Spain; 3 Nephrology Department. IIS-Fundación Jiménez Díaz, UAM. Av. Reyes Católicos, 2, 28040 Madrid, Spain

Correction to: *Scientific Reports* 10.1038/s41598-019-48426-4, published online 19 August 2019

In the original version of this Article, the author María Dolores Sánchez-Niño was incorrectly indexed. This error has now been corrected.

